# Supplementary material for: Influence of Breast Cancer Extracellular Vesicles on Immune Cell Activation: A Pilot Study
Source: Biology (Basel). 2023 Dec 15;12(12):1531. doi: 10.3390/biology12121531 (PMC10740516; doi:10.3390/biology12121531)
Supplement: Supplementary file 1 [file biology-12-01531-s001.zip › biology-2740033-supplementary.pdf]

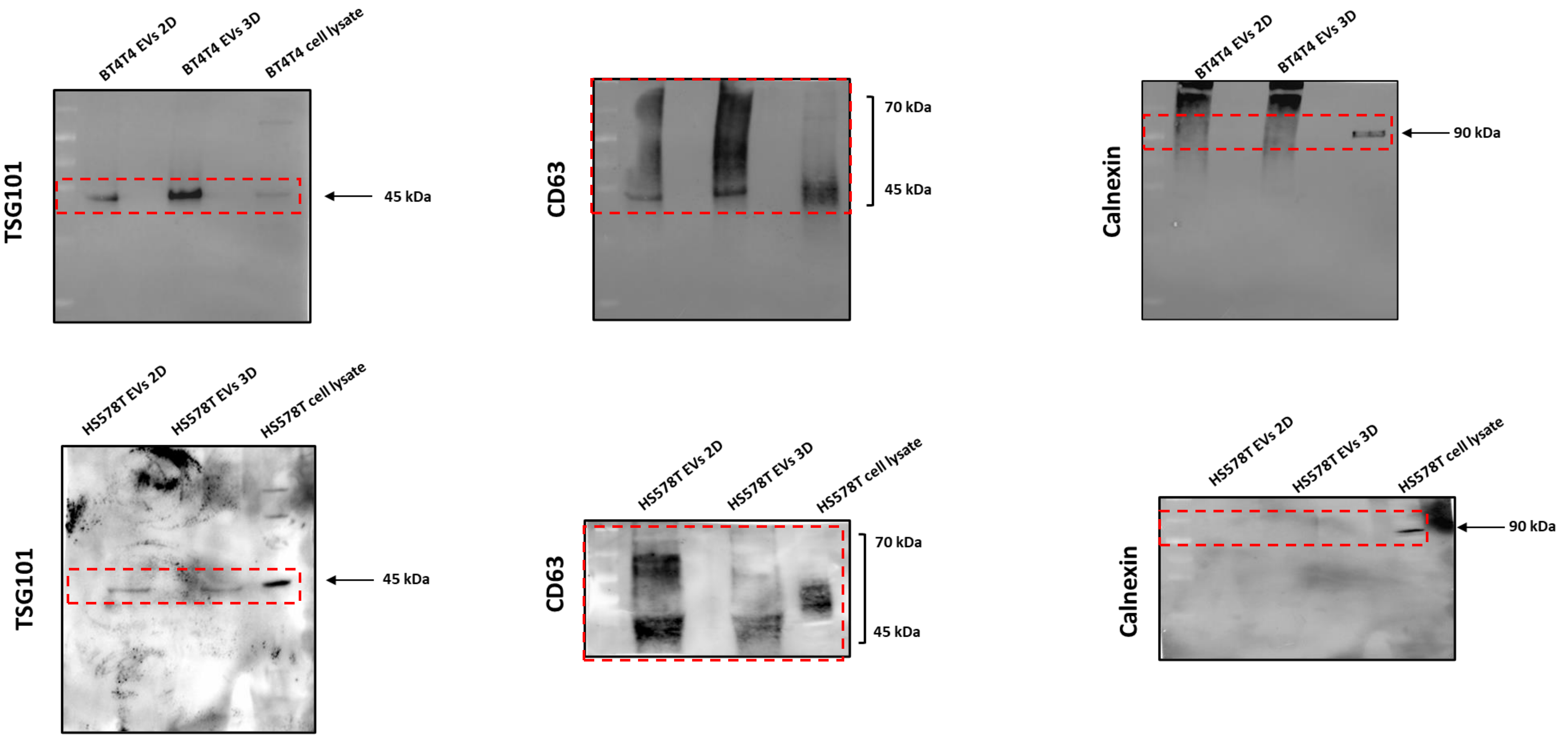

Supplementary Figure S1. Uncropped WB images reported in Figure1. Red boxs report the cropped WB inserted in Figure1.

HS578T 10k cells spheroid **BT474 10k cells spheroid**

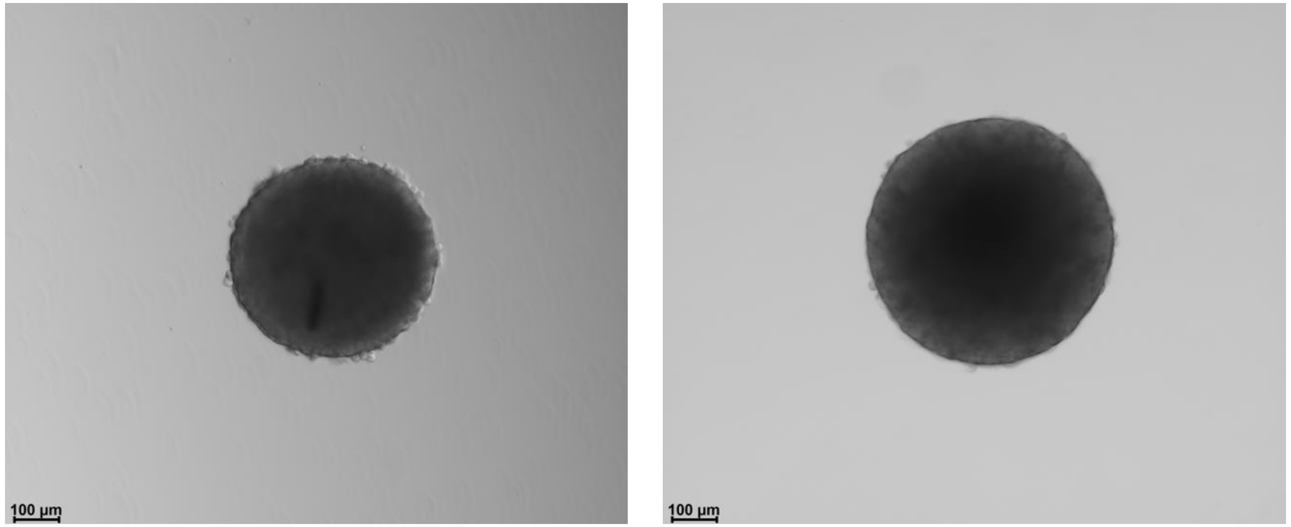

	Size
**HS578T spheroid**	460.2 µm
**BT474 spheroid**	588.8 µm

Supplementary Figure S2. Spheroids characterization. Representative confocal images of BT474 and HS578T spheroids and size (bottom table).

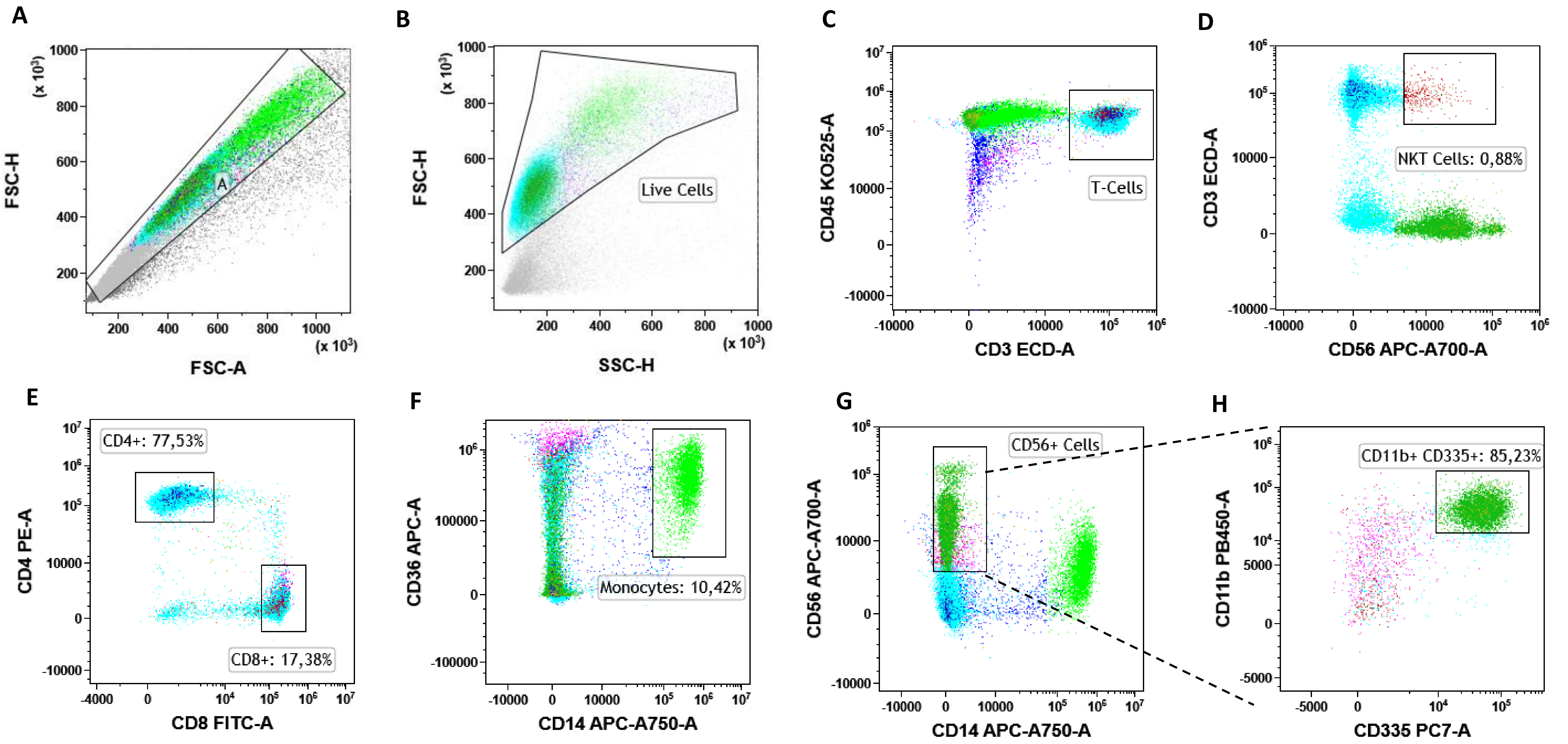

Supplementary Figure S3. Gating strategy of gated cells reported in Figure 2-3-4-5. Live cells (B) were gated using CD45+/CD3+ cells to detect T-cells (C). CD3+/CD56+ were selected as NKT Cells (D). Inside the T-Cells were separated CD4+ and CD8+ cells (E). From Live cells were identified the monocytes as CD14+ cells (F). From live cells, the NK cells were selected as CD56+ cells (G). Inside the CD56+, the CD11b+/CD335+ cells were selected (H).

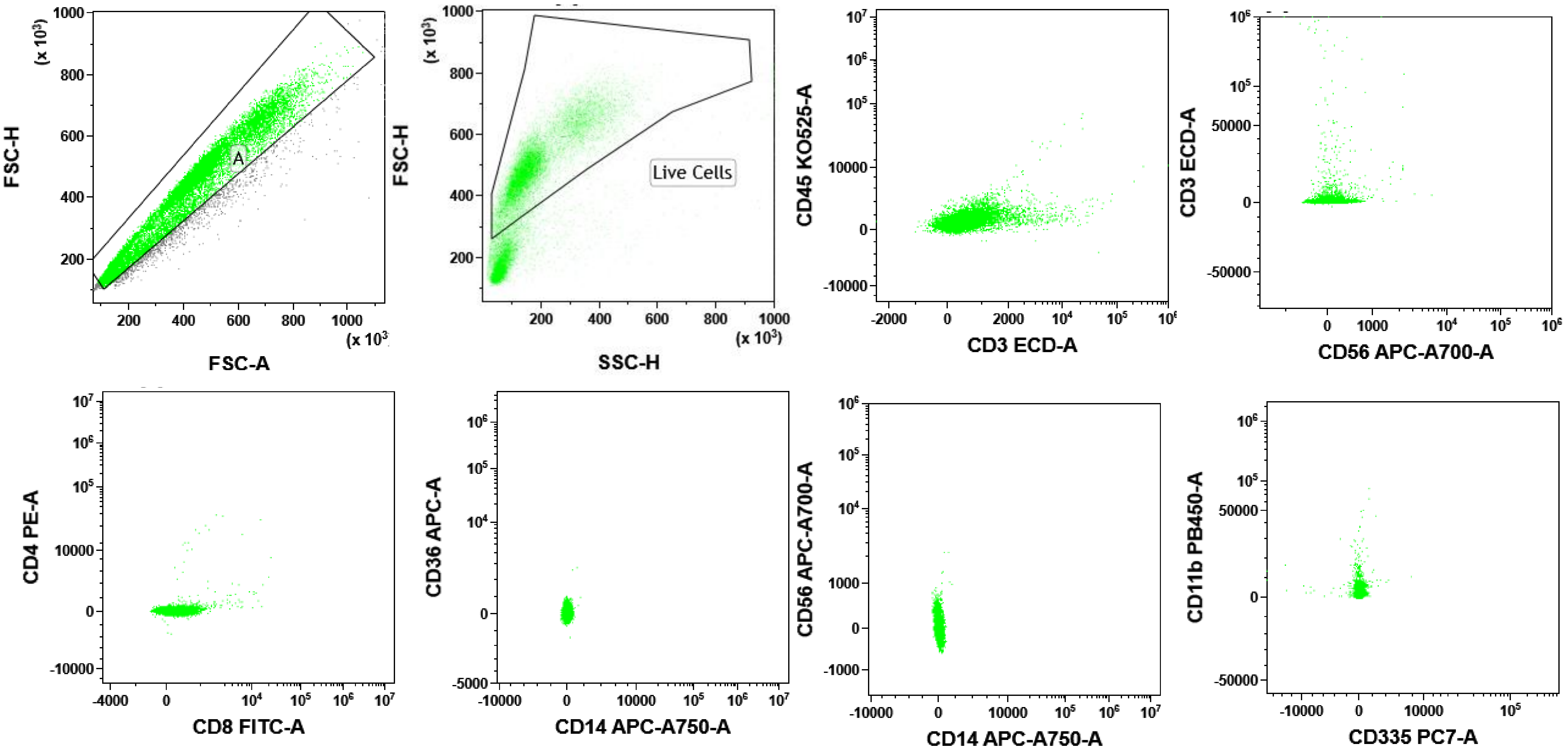

Supplementary Figure S4. Negative control of analyzed PBMCs.

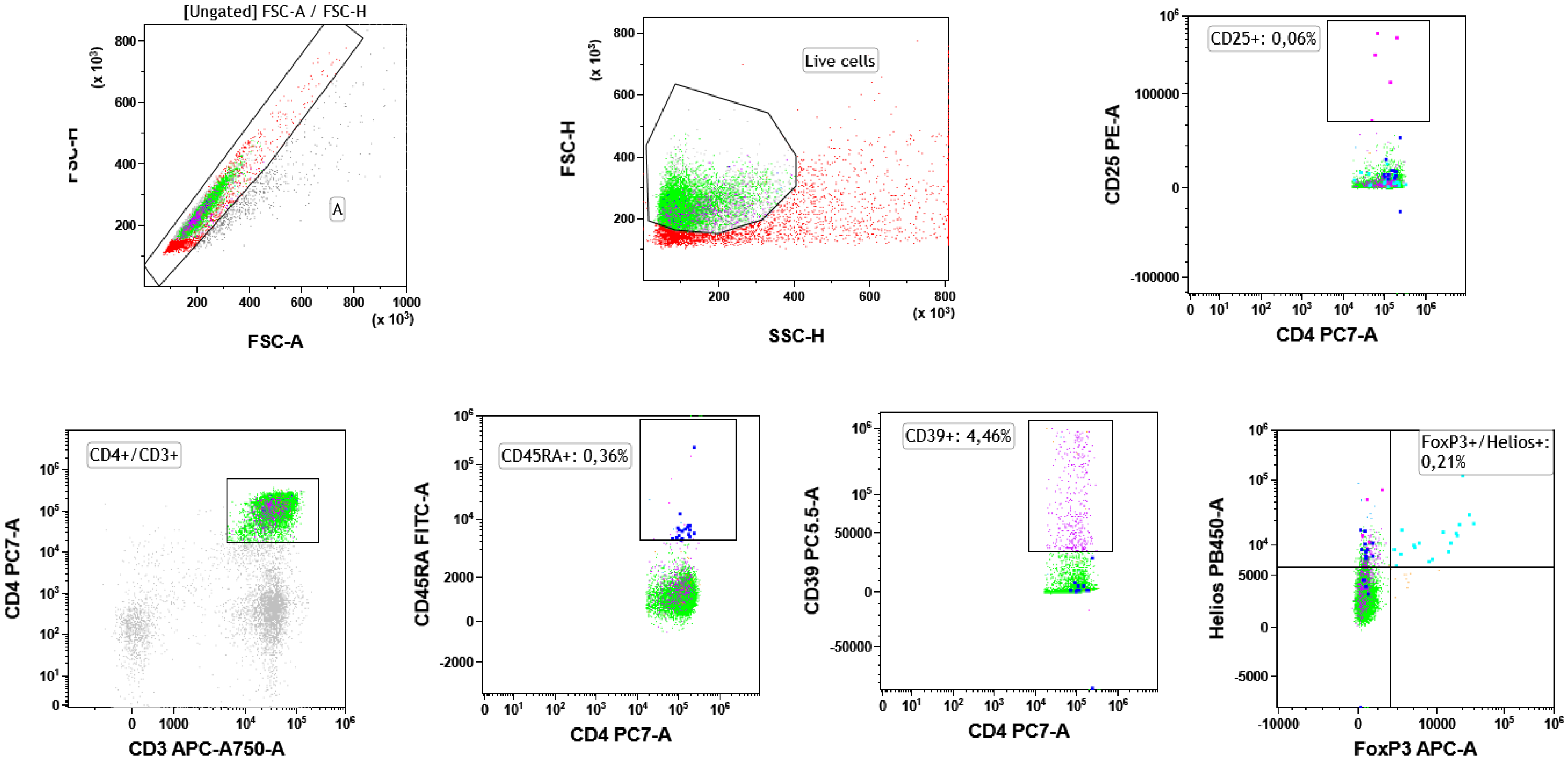

Supplementary Figure S5. Gating strategy of T-reg Cells identified in Figure 6.
